# Antidiabetic and Antioxidative Potential of the Blue Congo Variety of Purple Potato Extract in Streptozotocin-Induced Diabetic Rats

**DOI:** 10.3390/molecules24173126

**Published:** 2019-08-28

**Authors:** Paulina Strugała, Olha Dzydzan, Iryna Brodyak, Alicja Z. Kucharska, Piotr Kuropka, Mariana Liuta, Katarzyna Kaleta-Kuratewicz, Agnieszka Przewodowska, Dorota Michałowska, Janina Gabrielska, Natalia Sybirna

**Affiliations:** 1 Department of Physics and Biophysics, Wrocław University of Environmental and Life Sciences, C.K. Norwida 25, 50-375 Wrocław, Poland; 2 Department of Biochemistry, Ivan Franko National University of Lviv, 4 Hrushevskyi St., Lviv 79005, Ukraine; 3 Faculty of Biotechnology and Food Science, Department of Fruit, Vegetable and Plant Nutraceutical Technology, Wrocław University of Environmental and Life Sciences, J. Chełmońskiego 37/41, 51-630 Wrocław, Poland; 4 Faculty of Veterinary Medicine, Department of Biostructure and Animal Physiology, Wrocław University of Environmental and Life Sciences, C.K. Norwida 31, 50-375 Wrocław, Poland; 5 Plant Breeding and Acclimatization Institute-National Research Institute, Bonin Research Center, Bonin 3, 76-009 Bonin, Poland

**Keywords:** purple potatoes, Blue Congo, streptozotocin-induced diabetes mellitus, blood, antidiabetic activity, antioxidant enzymes, markers of oxidative stress

## Abstract

This study was designed to evaluate the effects of purple potato extract of the Blue Congo variety (PP) on diabetes and its antioxidant activities after two-week administration tostreptozotocin (STZ)-induced diabetic rats. The activities of PP were evaluated at a dose of 165 mg/kg body weight (b.w.) by estimating biochemical changes in blood plasma and through a histopathological study of kidney, muscles, and liver tissue. We evaluated the effect of treatment with extract on glucose level, glycated hemoglobin, activities of enzymatic antioxidants (including superoxide dismutase, glutathione peroxidase, and catalase), and lipid peroxidation. Moreover, we determined advanced glycation end-products (AGEs), advanced oxidation protein products (AOPPs), and the level of oxidative modified proteins (OMPs) as markers of carbonyl-oxidative stress in rats with diabetes. Using high-performance liquid chromatography, we identified five anthocyanins and six phenolic acids in the extract from Blue Congo with the dominant acylated anthocyanin as petunidin-3-*p*-coumaroyl-rutinoside-5-glucoside. The administration of Blue Congo extract lowered blood glucose, improved glucose tolerance, and decreased the amount of glycated hemoglobin. Furthermore, PP demonstrated an antioxidative effect, suppressed malondialdehyde levels, and restored antioxidant enzyme activities in diabetic rats. After administration of PP, we also noticed inhibition of OMP, AGE, and AOPP formation in the rats′ blood plasma.

## 1. Introduction

Diabetes mellitus (DM) is a severe metabolic disorder of multiple etiologies characterized by chronic hyperglycemia with disturbances of carbohydrate, lipid, and protein metabolism, resulting in defects in the action of insulin secretion. It is recognized as the wide-reaching disorder affecting people of almost all age groups [1]. Diabetes is divided into different types. Type 1 diabetes accounts for 5–10% of all diabetic cases. Its typical symptoms include chronic hyperglycemia and pathophysiology in the insulin production in β cells of the islets of Langerhans in the pancreas. Consequently, they lead to insulin deficiency. Type 2 diabetes is more frequent, accounting for 90–95% of all diabetic cases. It is characterized by insulin resistance and relative insulin deficiency, and it is frequently accompanied by overweight or obesity [2]. Until now, in medicine, no satisfactory therapy to cure DM was developed. The antidiabetic drugs address the symptoms of DM, not the causes that generate the disease. Currently, some synthetic drugs with anti-diabetic effects, including synthetic hypoglycemic agents like sulfonylureas groups, are being delivered orally. The therapeutic support in DM has several side effects such as weight gain, hypoglycemia, gastrointestinal disturbances, liver and kidney damage, and hypersensitivity reactions which can have a negative impact on the patient’s response to treatment [1,3]. The abovementioned side effects indicate that there is a need for further development of new, safer, and more effective oral antihyperglycemic agents for treating and managing diabetes complications, especially in long-term therapy. Medicinal plants are an important source for finding new remedies for health problems. Traditionally, numerous herbs are recommended in DM therapies. Additionally, the antidiabetic effects of many plants were reported by numerous researchers [3]. A number of recent reports indicated that the consumption of fruits and vegetables, especially rich in polyphenols, decreases the incidence of diabetes [4]. In recent years, experiments provided evidence that anthocyanins exert beneficial effects on the organism and may be helpful in preventing and treating some metabolic diseases, including diabetes [5].

Anthocyanins are hydrophilic compounds classified as flavonoids. They are naturally occurring colorants which are seen as the food pigments of primary importance in addition to chlorophyll, and they are responsible for the distribution of red, orange, blue, violet, and purple colors in fruits, flowers, and vegetables [6,7]. It is a generally accepted opinion that anthocyanins found in food do not show any toxic properties, even at high doses of those compounds [8,9]. Although anthocyanins are widely present in many fruits and vegetables, their main source is berries. According to recent reports, the presence of anthocyanin in colored potato and purple sweet potato is significant [10,11]. Potatoes are one of the most consumed vegetables in many countries and they are currently the fourth most important food crop worldwide after maize, wheat, and rice. The main difference between anthocyanins found in berries and those present in purple potatoes is that the latter create acylated and poly-glycosylated structures [12]. Acylation with various phenolic acids makes purple potato anthocyanins unique due to the fact that they show higher molecular stability, e.g., they provide advantages toward oxygen, pH, heat, and light resistance, and enzyme sensitivity [13,14].

Previous studies showed that anthocyanins present in purple potatoes possess many biological activities, including antioxidative [15,16], anti-atherosclerotic [17], antimicrobial [10], anti-mutagenic [16,18], and hepatoprotective activity [19]; moreover, these compounds can protect against oxidative stress damage [20]. In addition, Zhao et al. [16] showed that extracts from purple sweet potato inhibited Sarcoma S180 cell growth in ICR mice. Moreover, in studies carried out by Lim et al. [21], based on in vitro and in vivo experiments, it was suggested that anthocyanin-enriched sweet potato extract may protect against colorectal cancer by inducing cell-cycle arrest, as well as antiproliferative and apoptotic mechanisms.

Purple potatoes can be used as novel sources of natural colorants and antioxidants for human health, e.g., in managing hyperglycemia. This study, thus, aimed to demonstrate the anti-diabetic activity and antioxidant status of purple potato extract from the Blue Congo variety (PP) in Wistar rats with diabetes induced by streptozotocin. We evaluated the effect of treatment with extract on glucose level, glycated hemoglobin, activities of enzymatic antioxidants (superoxide dismutase, glutathione peroxidase, and catalase), and lipid peroxidation. Moreover, we determined advanced glycation end-products, advanced oxidation protein products, and the level of oxidative modified proteins as markers of carbonyl-oxidative stress in rats with DM. Finally, the histopathological studies of kidney, muscles, and liver tissue are also presented. According to the best of our knowledge, this is the first report to show in vivo the antihyperglycemic effects of purple potatoes of the variety Blue Congo variety.

## 2. Results and Discussion

One of the commonly employed methods used to induce type 1 DM is intraperitoneal administration of streptozotocin (STZ) to experimental animals. Therefore, we evaluated the antidiabetic and antioxidant activity of Blue Congo extract referring to this model. Blood glucose levels are mainly controlled by insulin secretion from pancreatic cells. STZ acts as a nitrosourea analogue and destroys pancreatic β cells in a variety of ways. The selective pancreatic β-cell toxicity and the diabetic conditions resulting from STZ induction are related to the glucose moiety in its chemical structure which enables STZ to penetrate into the β cell via the low-affinity glucose 2 transporter in the plasma membrane. This is due to the fact that β cells of the pancreas are more active than other cells in taking up glucose and, consequently, they are more sensitive than other cells to STZ challenge [22]. As a result, it is possible to explain why experimental animals induced with STZ tend to have renal and liver damage [23]. The mechanism of STZ-induced diabetes in rats mainly involves DNA alkylation (forming methyl nitrosourea moiety), generation of reactive oxygen species (ROS; superoxide radicals, hydrogen peroxide, and hydroxyl radicals), and enhanced formation of nitric oxide (NO) in β cells of the pancreas (inhibiting aconitase activity and participating in DNA damage) [24,25]. The generation of ROS and the subsequent increase in local oxidative stress, DNA methylation, and protein modification are explained as the pathophysiological mechanisms of STZ-induced diabetes [26]. Hence, the essential criteria for the treatment of diabetes involve scavenging free radicals, while maintaining blood glucose and lipid levels close to normal [27].

Thus, anthocyanins from purple potatoes are considered to be promising agents against STZ-induced diabetes since the oxidative stress can be diminished via inhibiting ROS generation, protein oxidation, and lipid peroxidation. A review of the literature shows that a scientific evaluation is yet to be conducted to check the antidiabetic potential of purple potato extract of the Blue Congo variety.

### 2.1. Phenolic Content Determined by HPLC/LC–MS Method

Using the HPLC/LC–MS chromatographic method, we performed a quantitative and qualitative analysis of the Blue Congo extract compounds. The results are presented in Table 1. The compounds were identified by their retention times, elution order, spectra of individual peaks (ultraviolet/visible light (UV/Vis), MS, MS/MS), and by comparison with literature data [28,29]. The content of phenolic compounds was expressed as mg/g of preparation, found to be 237.07 mg per gram dry matter (d.m.). In the extract, we determined 11 main compounds from two groups: anthocyanins (five compounds) and phenolic acids (six compounds). The basic structure of the anthocyanins contained acylated and glycosidic forms with phenolic acids, as well as glucose and rutinose. Using electrospray ionization (ESI) MS, we determined in the Blue Congo the concentrations of aglycone ions: petunidin (*m/z* = 317), delphinidin (*m/z* = 303), and malvidin (*m/z* = 331). Petunidin is commonly believed to be the most abundant aglycone with the total amount of its derivatives accounting for up to 90.5% of total anthocyanins. This discovery is in line with the results obtained in previous studies [30]. We identified the presence of acylated anthocyanins such as petunidin-3-*O*-*p*-coumaryl-rutinoside-5-*O*-glucoside, petunidin-3-*O*-*p*-caffeyl-rutinoside-5-*O*-glucoside, delphinidin-3-*O*-*p*-coumaroyl-rutinoside-5-*O*-glucoside, and malwidin-3-*O*-*p*-coumaryl-rutinoside-5-glucoside, and one non-acylated anthocyanin, petunidin-3-*O*-rutinoside-5-*O*-glucoside. It should be stressed that caffeic and *p*-coumaric acids were found as acyl residues in the acylated structure. Petunidin-2-*p*-coumaryl-rutinoside-5-glucoside, as the main compound, covered almost 74% of the total anthocyanin content in the Blue Congo extract. Our results are in line with other reports [31,32] which found the same dominant acylated anthocyanins in Blue Congo potatoes as petunidin derivatives. Also, Hillebrand et al. [33], using the HPLC/ESI-MS method, found that three varieties of purple potatoes contained one major acylated anthocyanin that was petunidin-3-*p*-coumaroyl-rutinoside-5-glucoside. Among the group of six main phenolic acids, the following were identified: neochlorogenic, chlorogenic, and cryptochlorogenic acids (*m/z* = 191). In the group of phenolic acids, chlorogenic acid was dominant, accounting for up to 59%.

### 2.2. Effect of Purple Potato (PP) Extract from Blue Congo Variety on Glucose-Related Parameters in STZ-Induced Diabetic Rats

**Figure 1:**
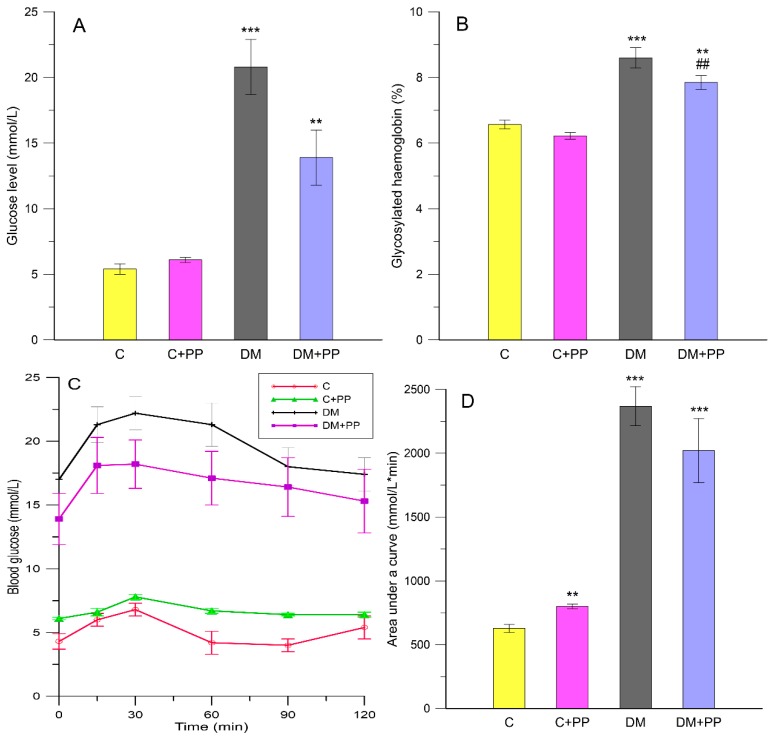
Glucose-related parameters under administration of purple potato (PP) extract from Blue Congo variety: (**A**) fasting blood glucose; (**B**) glycated hemoglobin, % of total hemoglobin; (**C**) oral glucose tolerance test; (**D**) area under the curve after glucose load of rats. C—control group, C + PP—control rats receiving extract from Blue Congo (daily dose of 165 mg/kg body weight (b.w.) for 14 days), diabetes mellitus (DM)—diabetic group, DM + PP—diabetic rats treated with Blue Congo extract (daily dose of 165 mg/kg b.w. for 14 days). Results are shown as means ± SD. Significance was measured using one-way analysis of variance (ANOVA). Designations: ** *p* < 0.01, *** *p* < 0.001 compared to the control group; ## *p* < 0.01 compared to the DM group.

The fasting blood glucose in STZ-induced diabetic rats (DM groups) was significantly higher (*p* < 0.001) than that in control rats (Figure 1A). After two weeks of supplementation with the PP extract, a decrease in fasting blood glucose concentration in group DM + PP (13.9 ± 2.1 mmol/L) was observed in comparison to the diabetic group (20.8 ± 2.1 mmol/L) (Figure 1A). The results indicate that the Blue Congo extract induced a hypoglycemic effect in STZ-treated diabetic rats. According to Matsui et al. [34], both the diacylated anthocyanin which was isolated from purple sweet potatoes (*Ipomoea batatas* cv. *Ayamurasaki*) and the extract possess a postprandial anti-hyperglycemic effect in rats. Furthermore, according to Zhao et al. [16], the extract from the same variety of sweet potatoes significantly reduced plasma glucose levels in STZ-treated mice.

The process of glycosylated hemoglobin (HbA1c) production is based on the non-enzymatic glycosylation of hemoglobin. This compound is produced under chronic hyperglycemia and oxidative stress, steadily and uninterruptedly within the whole red blood cell (RBC) lifespan, and it is fully independent of a broad scope of factors including diet, insulin, or exercise on the day when the test is conducted. As a result, HbA1c plays a role as a marker in glycemic control and, consequently, it is important in diagnosing diabetes and diabetic complications [24]. STZ-induced diabetes was demonstrated to induce an increase in HbA1c levels [2]. In our study, STZ-induced diabetic rats showed an increase in HbA1c levels (about 24% compared to the control group) (Figure 1B). After treatment with PP extract, HbA1c levels in erythrocytes were reduced (about 12% compared to the DM group, *p* < 0.01), implying that purple potatoes may ameliorate oxidative damage caused by the glycation reaction in diabetes. A similar effect was demonstrated in studies by Sabahiet et al. [35] and Grace et al. [36], in which anthocyanin fractions from *Berberis integerrima* fruit and from lowbush blueberry reduced glucose in treated animals compared to the diabetic group.

Oral glucose tolerance tests (OGTTs) were used to determine the acute effect of the PP extract on diabetic rats. Figure 1C shows the OGTT test results. The blood glucose in all groups reached its peak value in 30 min. The PP extract supplementation was found to improve glucose tolerance (Figure 1C). Indeed, the glycemic response to the OGTT, assessed by the area under the curve for blood glucose (AUC_glc_), was shown to be lower in the PP extract-treated diabetic rats (Figure 1D) than in the non-treated diabetic group (about 15%). The PP extract affected fasting blood glucose and the postprandial glucose level. Glucose concentration in blood decreased when PP was administrated to diabetic rats. Previous studies confirmed the reduction of blood glucose levels using compounds present in colored potatoes in an animal model. For instance, Jang et al. [37] showed that oral treatments with cyanidin 3-caffeoyl-*p*-hydroxybenzoyl-sophoroside-5-glucoside (purified from purple sweet potatoes—Korean variety Shinzami) in mice significantly reduced fasting blood glucose.

### 2.3. The Effect of PP Extracts on Erythrocyte- and Leukocyte-Related Parameters

In the diabetes group, the total count of erythrocytes and hemoglobin concentration did not change significantly, but the mean cell hemoglobin (MCH) and color index decreased by 20% compared to the control group (Table 2). After supplementing diabetic rats with the PP extract, the total count of erythrocytes and hemoglobin concentration did not change significantly compared to the non-treated diabetic group. However, the MCH and color index increased to control values after treatment with PP extract. Counted leukocytes did not reveal differences in diabetic rats (DM) and in the PP-treated diabetic group (DM + PP) when compared to the control group (C) (Table 2). However, the content of leukocytes in the PP-treated control group (C + PP) increased by 38% compared to the control group (*p* < 0.01).

### 2.4. Measurement of Oxidative Stress-Related Enzymes in Leukocytes

Oxidative stress occurs when free-radical production exceeds the antioxidant capacity of a cell. Most of the radicals are ROS, such as hydroxyl radicals, hydrogen peroxide, and superoxide anions, all of which can damage crucial cellular compounds, such as lipids, carbohydrates, proteins, and DNA. The human body possesses antioxidant defense systems which deal with ROS, including the antioxidant enzymes superoxide dismutase (SOD), glutathione peroxidase (GPx), and catalase (CAT), and non-enzymatic antioxidants such as reduced glutathione (GSH) [38]. In the process of development of diabetes, oxidative stress is, according to many studies, one of the most important factors [39,40].

**Figure 2:**
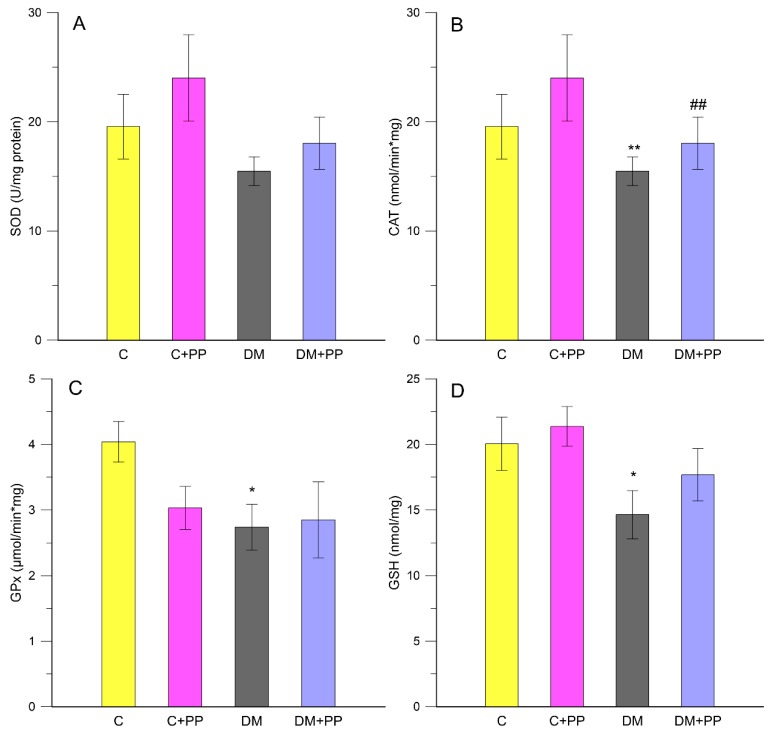
Effects of purple potato (PP) extract from Blue Congo (daily dose of 165 mg/kg b.w. for 14 days) on activity of antioxidant enzymes in leukocytes: (**A**) SOD, superoxide dismutase; (**B**) CAT, catalase; (**C**) GPx, glutathione peroxidase, (**D**) GSH, reduced glutathione. C—control group, C + PP—control rats receiving extract from Blue Congo, DM—diabetic group, DM + PP—diabetic rats treated with Blue Congo extract. Data are presented as means ± SD. Significance was measured using one-way analysis of variance (ANOVA). Designations: * *p* < 0.05, ** *p* < 0.01 compared to the control group; ^##^ *p* < 0.01 compared to the DM group.

To investigate the protective systems of PP extract against oxidative stress in diabetic conditions, animals of control and diabetic groups were administered PP extract for 14 days; then, we determined the levels of various oxidative stress parameters in their leukocytes. The activities of enzymatic antioxidants (SOD, CAT, and GPx) were examined in the leukocytes of each group. As shown in Figure 2A–C, the diabetic group showed a decrease in SOD, CAT, and GPx activities compared to control. After the PP treatment of animals with diabetes, the activities of SOD, CAT, and GPx increased compared to those of the non-treated diabetic group. 

SOD catalyzes the dismutation of superoxide anion free radicals (O_2_^−^) into molecular oxygen and hydrogen peroxide (H_2_O_2_), and decreases O_2_^−^ levels responsible for damaging the cells when at an excessive value. The SOD activity was found to be lower in diabetic subjects [40]. SOD activity was lower in the DM groups by about 26% compared to the control rats, whereas there was no significant change in the DM + PP group compared to the non-treated diabetic rats (about 14%) (Figure 2A).

CAT is a hemeprotein, which is present in virtually all mammalian cells, and it catalyzes the decomposition of hydrogen peroxide to water and oxygen [41]. CAT activity was significantly (*p* < 0.01) lower in the diabetic rats by about twofold compared to the control rats, whereas it was significantly (*p* < 0.01) higher (about 74%) in the DM + PP group than in the non-treated diabetic rats (Figure 2B).

Glutathione peroxidase plays a critical role in the reduction of lipid and hydrogen peroxides. If GPx activity is decreased, more hydrogen peroxide is present, which leads to direct tissue damage and the activation of nuclear factor-κB-related inflammatory pathways. GPx catalyzes the reaction of hydroperoxides with GSH to form glutathione disulfide. Decreased GPx activity could be explained by the excess of ROS present in diabetic rats [42]. The decrease may also be explained by the GSH decreased availability which was shown to be depleted in diabetes [40]. From our experimental studies, diabetic rats exhibited reduced activity of GPx by 1.4-fold (2.74 ± 0.35 µmol/min × mg), (*p* < 0.05) compared to the control rats (4.04 ± 0.31 µmol/min × mg), (Figure 2C). The supplementation of PP extract slightly increased GPx activity (2.85 ± 0.58 µmol/min × mg) in the diabetic rats.

GSH is one of the essential antioxidants for maintaining cell integrity against ROS, as it can scavenge free radicals and reduce H_2_O_2_. The depletion of GSH below its basal level promotes the generation of ROS and oxidative stress with a cascade of effects on the functional and structural cell integrity and organelle membranes. Studies showed that the GSH concentration in diabetic rats is significantly lower when compared to control rats [40]. In our studies, a decreased level of GSH in the DM group (14.64 ± 1.83 nmol/mg) was observed in comparison to the control rats (20.04 ± 2.03 nmol/mg), while the treatment with PP extract increased the level of GSH in the DM + PP group (17.68 ± 1.99 nmol/mg) (Figure 2D). Our results are similar to those of Chen et al. [39], who indicated that anthocyanin extracts from black soybean possess antioxidant capacity, thereby significantly increasing the activities of SOD, CAT, and GPx and decreasing malondialdehyde (MDA) levels in STZ-induced diabetic mice. In addition, Kruger et al. [43] revealed that grape and black rice anthocyanins could effectively limit oxidant stress in vitro and in vivo.

### 2.5. The Effect of PP Extracts on Lipid Peroxidation in Blood Plasma of Rats

**Figure 3:**
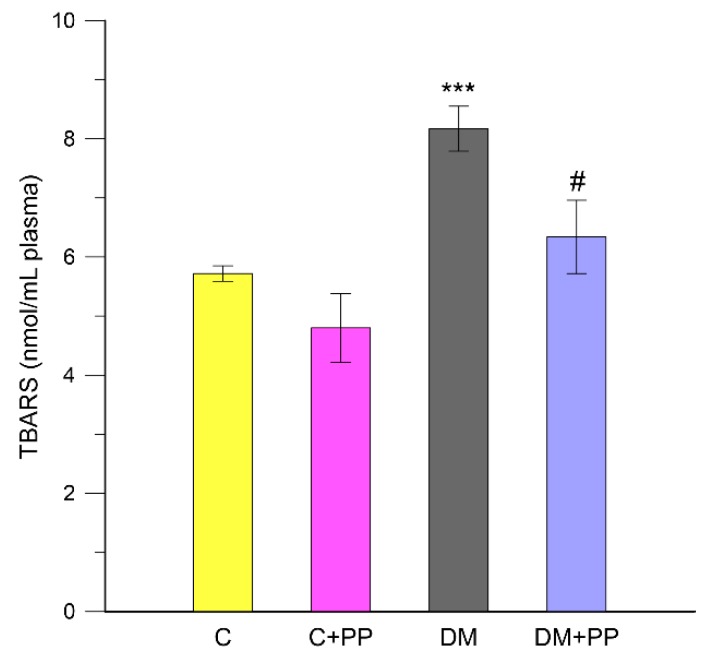
Effects of purple potato (PP) extract from Blue Congo (daily dose of 165 mg/kg b.w. for 14 days) on the content of thiobarbituric acid (TBA)-reactive substances (TBARS) of lipid peroxidation (LPO) in the blood plasma of rats. C – control group, C + PP—control rats receiving extract from Blue Congo, DM—diabetic group, DM + PP—diabetic rats treated with Blue Congo extract. The results are shown as means ± SD. Designations: *** *p* < 0.001 compared to the control group; # *p* < 0.05 compared to the DM group.

Streptozotocin induces severe oxidative stress in diabetic animals and possibly induces the peroxidation of polyunsaturated fatty acids, leading to the formation of MDA, which is a by-product of lipid peroxidation [44]. Therefore, lipid peroxidation is considered to be an important parameter of oxidative stress. In the present study, we measured lipid peroxidation in thiobarbituric acid (TBA)-reactive substances (TBARS) test. Figure 3 shows the levels of malondialdehyde in the blood plasma of each experimental group rats. 

Diabetic rats showed significantly (*p* < 0.001) elevated levels of lipid peroxidation in the plasma by 30% compared with that in healthy rats (control groups). The observed increase in the level of TBARS in diabetic rats is generally thought to be a consequence of increased production and liberation into the circulation of tissue lipid peroxides due to pathological changes [41]. Supplementation of PP extract significantly reduced the levels of MDA by 22% compared with that in diabetic rats (*p* < 0.05). The results were consistent with a previous study in which the treatment of STZ-induced diabetic rats with anthocyanins from black soybean seed coats resulted in a marked decrease in MDA levels [26]. Moreover, the antioxidative properties of purple sweet potatoes to prevent many complications in DM were proven in animals. To illustrate this opinion, Satriyasa [45] indicated that the MDA level in STZ-induced diabetic rats can be reduced by administration of an aqueous extract of Balinese purple sweet potato tubers for 60 days.

### 2.6. Effects of PP on the Content of Carbonyl-Oxidative Stress Metabolites in Rats′ Blood Plasma

In diabetes, hyperglycemia, protein glycation, and glucose auto-oxidation are believed to induce free-radical generation [46]. Among the major markers used for estimation of oxidative damage of proteins, there are carbonyl derivatives, 3-nitrotyrosine, *S*-nitrotriazole, 3-chlorotyrosine, bromotyrosine, methionine sulfoxide, dityrosine, oxo-histidine, and the so-called advanced products of protein oxidation [47]. Stable carbonyl derivatives that accumulate in the organism constitute valuable markers that allow determining the intensity of protein oxidation processes.

**Figure 4:**
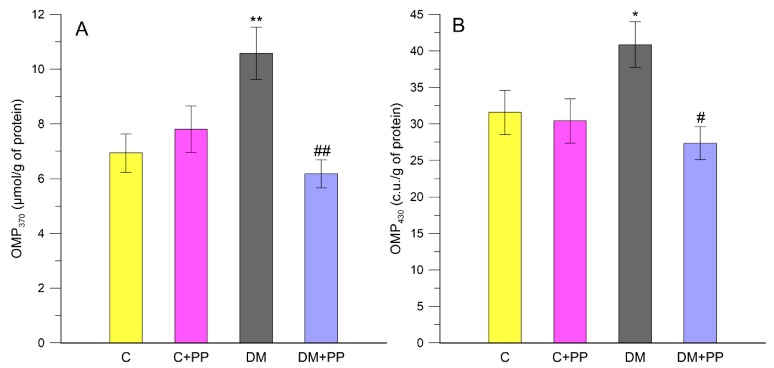
Oxidative modified proteins (OMP) of neutral (**A**) and basic (**B**) characteristics in rats′ blood plasma. C—control group, C + PP—control rats receiving extract from Blue Congo (daily dose of 165 mg/kg b.w. for 14 days), DM—diabetic group, DM + PP—diabetic rats treated with Blue Congo extract (daily dose of 165 mg/kg b.w. for 14 days). The results are shown as means ± SD. Designations: * *p* < 0.05, ** *p* < 0.01 compared to the control group; # *p* < 0.05, ## *p* < 0.01 compared to the DM group.

In our study, it was found that the level of protein carbonyl groups (oxidative modified proteins OMP_430_ and OMP_370_) as biomarkers of oxidative stress increased by 34.4% and 22.7%, respectively, in rats′ blood plasma under diabetic (DM group) conditions compared to control (*p* < 0.05). The administration of PP caused a significant decrease in the level of neutral and basic OMPs by 41.6% and 33.1%, respectively, compared to the DM group (*p* < 0.01) (Figure 4A,B).

**Figure 5:**
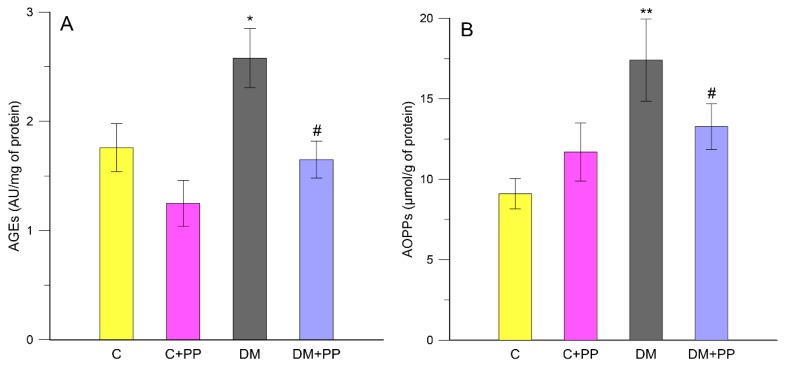
The effect of purple potato (PP) extract from Blue Congo (daily dose of 165 mg/kg b.w. for 14 days) on the content of advanced glycation end-products (AGEs) (**A**) and advanced oxidation protein products (AOPPs) (**B**) in rats′ blood plasma. C—control group, C + PP—control rats receiving extract from Blue Congo, DM—diabetic group, DM + PP—diabetic rats treated with Blue Congo extract. The results are shown as means ± SD. Designations: * *p* < 0.05, ** *p* < 0.01 compared to the control group; # *p* < 0.05 compared to the DM group.

Free-radical oxidative damage leads to losses in specific protein function. Since proteins have varied biological functions, there are often unique functional consequences resulting from their modification. It is estimated that almost every third protein in a cell of older animals is dysfunctional as an enzyme or structural protein due to oxidative damage [48]. The advanced oxidation protein products (AOPPs) are mainly derivatives of oxidatively modified albumin, but also those of fibrinogen and lipoprotein that arise due to intense oxidative stress. The AOPPs are taken as markers of protein oxidative damage and inflammation reaction. In the body, AOPPs are created throughout life, with the intensity slightly rising with aging. Their significant increase was found in many pathological states, including diabetes. In diabetes, the production of AOPPs is induced by the increased carbonyl-oxidation processes, disturbances of the oxidation–antioxidation balance, and the co-existent inflammation states [40,49]. We found an elevation of plasma AOPPs in rats with DM compared with the control rats of about 48% (*p* < 0.05) (Figure 5B). The plasma AOPPs levels were not significantly increased (*p* > 0.05) in the control rats receiving extract from Blue Congo as compared to the control rats (Figure 5B). In contrast, the administration of PP extract to rats with DM caused a significant decrease in this indicator compared to the non-treated diabetic group by about 23.7% (*p* < 0.05). In DM patients, it is reported that hyperglycemia can promote ROS and reactive nitrogen species (RNS) accumulation through different metabolic pathways which increase the formation of advanced glycation end-products (AGEs) [41]. Excessive availability of glucose promotes enhanced production of AGEs [50]. AGE formation and accumulation increasingly occurs under diabetic conditions, and, even if glycemic control is restored, AGEs can remain in tissues of diabetic subjects for a long time. In our study, the plasma AGE level was significantly increased by about 31.8% in the diabetic group compared to the control rats (*p* < 0.05) (Figure 5A). After treatment of the diabetic rats with PP extract, the AGE level was significantly lowered compared to the non-treated diabetic group by about 36% (*p* < 0.05). In the present study, significant increases in AOPPs and AGEs were found in diabetic rats, which is concurrent with the study of Kalousova et al. [49], which demonstrated significantly elevated levels of AOPPs in patients with both types of DM. Taken together, the present data depicted the capability of Blue Congo extract to restore the overall balance between oxidative and antioxidative systems in the rats with experimental diabetes.

We speculate that the indicated antidiabetic and antioxidant activities of Blue Congo extract are related to its anthocyanin content, yet it seems that phenolic acids also possess a strong capacity to act as antioxidants in diabetes, as indicated by numerous scientific reports. For instance, Roy et al. [46] reported that pelargonidin strengthened the antioxidant defense systems through increased activity of antioxidant enzymes, such as SOD and CAT, and effectively reverted serum MDA to normal levels of the STZ-induced diabetic rats. Valcheva-Kuzmanova et al. [51] showed that fruit juice from chokeberry significantly decreased the STZ-induced abnormalities in blood glucose and triglycerides in diabetic rats. The protective effects of anthocyanins and berries were well documented in the review article by Belwal et al. [52]. Other than berries, anthocyanins present in vegetables such as sweet potatoes, purple corn, and black soybean were also tested in vivo against diabetic, insulin resistance, weakened antioxidant defense system, and obesity, and they were found to be effective [26,34,53]. On the other hand, many studies suggested that phenolic acids, especially chlorogenic acid, have antidiabetic effects [17]. Chlorogenic acid suppresses postprandial hyperglycemia by inhibiting α-glucosidase inhibitors [54]. In addition, chlorogenic acid was proven to have anti-hyperglycemic effects in humans [55]. One of the potential mechanisms from using anthocyanins in diabetes is based on increasing glucose transporter 4 (GLUT-4) translocations. Another type of therapy is based on the observation that anthocyanins help to raise the phosphorylation of AMP-activated protein kinase (AMPK) and induce enzyme activation [35,52]. Moreover, it seems to be likely that one of the indirect protective mechanisms that affects cell integrity is the mechanism associated with nuclear respiratory factor 1 (Nrf1) activation [56]. Unfortunately, further studies are required due to the lack of scientific publications referring to this problem.

### 2.7. Histopathology Study

The chronic diabetes that characterizes this disorder results from defects in insulin secretion; it is also associated with long-term damage, dysfunction, and failure of various organs such as pancreas, kidneys, liver, eyes, heart, and blood vessels [1].

#### 2.7.1. Changes in Muscle Tissue 

**Figure 6:**
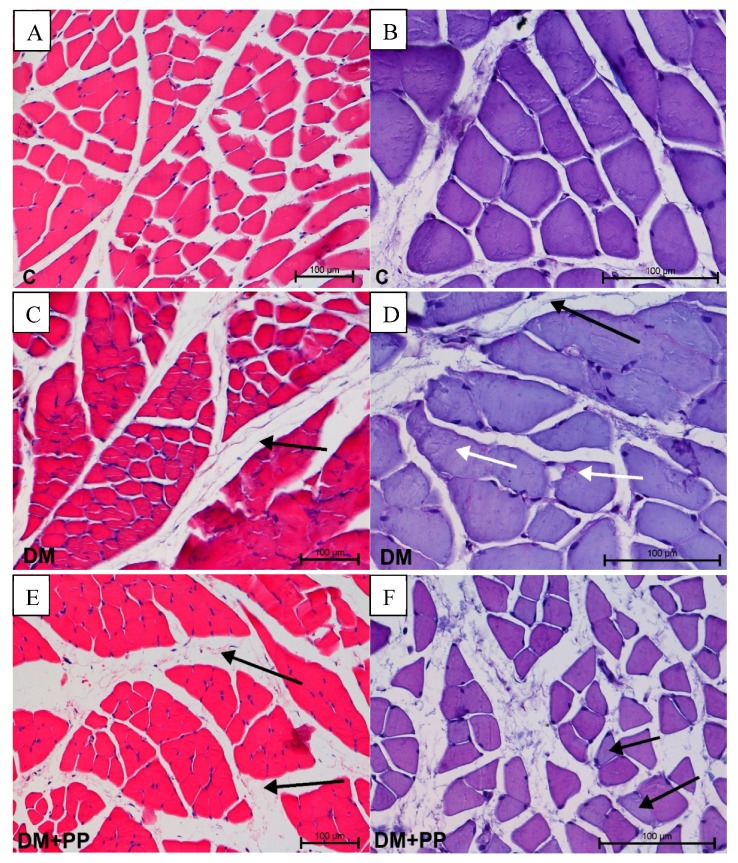
Histopathological studies of Blue Congo extract on the muscle tissue of streptozotocin (STZ)-induced diabetic rats. Note the presence of edema of connective tissue in the DM and DM + PP groups (arrow); photos on from the left side (**C**,**E**). Different periodic acid–Schiff (PAS)-positive reaction (white arrows) in myocytes (**D**,**F**). Photos on left side stained with hematoxylin and eosin (H&E), magnification 200× (**A**,**C**,**E**); photos on right side show PAS-positive staining, magnification 400× (**B**,**D**,**F**). C—control rats (healthy rats), DM—diabetic rats, DM + PP—diabetic rats treated with Blue Congo extract for 14 days (at a dose of 165 mg/kg b.w./day).

The muscles histology results showed that the changes concerned the localization of the periodic acid–Schiff (PAS)-positive reaction, as well as the presence of edema. Edematous changes in connective tissue surrounding muscle fibers in the DM and DM + PP group were observed (Figure 6C,E). No visible changes in fibroblast number or infiltrations of leukocytes were found. In the diabetic group, a PAS-positive reaction was noted in sarcolemma and as aggregates in the sarcoplasm of myocytes (Figure 6D). In the DM + PP group, the PAS-positive reaction was extremely irregular in the cells (Figure 6F). In some cases, the reaction was very strong and, in others, very weak. Morphometrical analysis did not reveal any differences in cell size.

#### 2.7.2. Changes in Kidney

**Figure 7:**
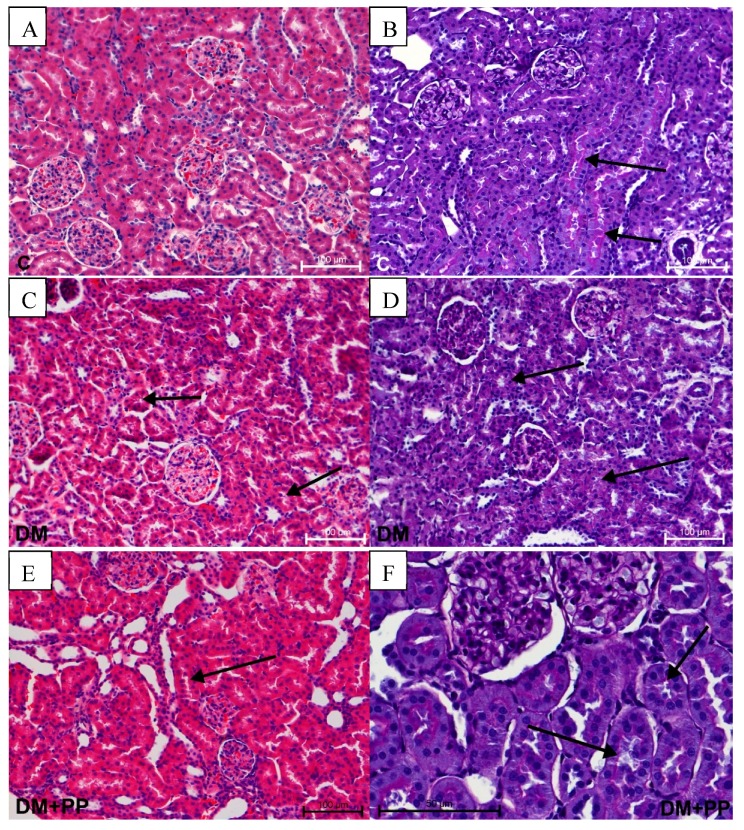
Histopathological studies of Blue Congo extract on the kidney tissue of STZ-induced diabetic rats. Note the presence of increased diuresis in proximal tubules (arrows) in the DM and DM + PP group photos on the left side (**C**,**E**). Different PAS-positive reaction (pink color) in proximal tubules (arrows), distal tubules, and capillary tuft (**D**,**F**). Photos on left side stained with H&E, magnification 200× (**A**,**C**,**E**); photos on right side show PAS-positive staining, magnification 200× (**B**,**D**), magnification 400× (**F**). C—control rats (healthy rats), DM—diabetic rats, DM + PP—diabetic rats treated with Blue Congo extract for 14 days (at a dose of 165 mg/kg b.w./day).

One of the most serious complications caused by increased glucose content in the blood is diabetic nephropathy. There was increased diuresis in the DM and DM + PP group with an enlarged lumen of proximal and distal tubules (especially in DM group) (Figure 7C). The lumen of tubules is irregularly enlarged upon specifically changing the structure of the cuboidal epithelium. In the DM + PP group, a PAS-positive reaction was noted in the basal membrane, as well as in the brush border on the apical part of epithelium in the proximal tubules and in the capillary tuft of the glomerulus (Figure 7F). A regular arrangement of PAS-positive reaction was noted in the control group (Figure 7B). No degeneration or inflammatory processes were visible. In the groups treated with Blue Congo extract, the diabetic nephropathy of rats was alleviated to a significant extent.

#### 2.7.3. Changes in Liver

**Figure 8:**
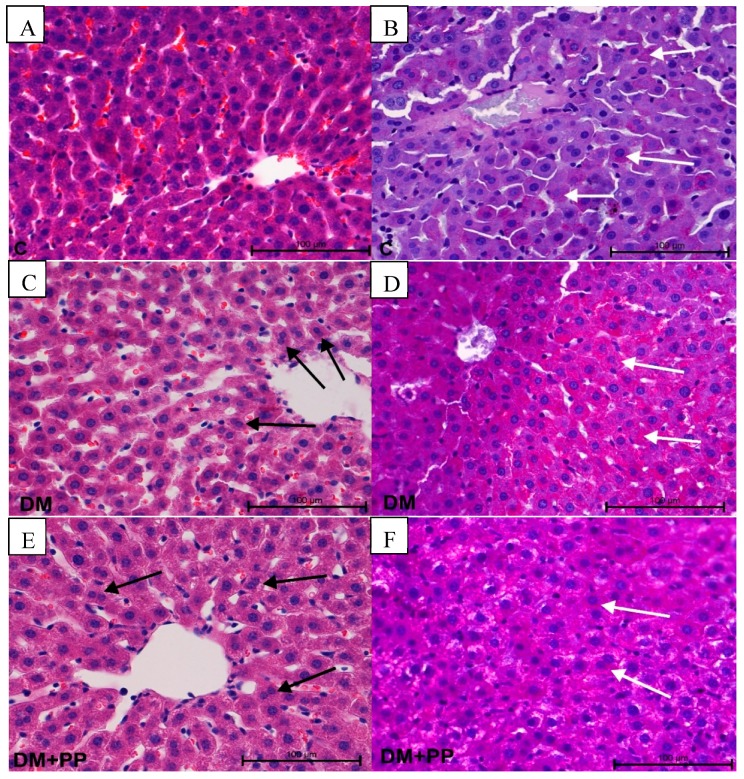
Histopathological studies of Blue Congo extract on the liver tissue of STZ-induced diabetic rats. Note the presence of increased number of hepatocytes with two nuclei (arrows) in the DM and DM + PP group on the left side (**C**,**E**). Different PAS-positive reaction (pink color) in hepatocytes (white arrows) (**D**,**F**). Photos on left side stained with H&E, magnification 200× (**A**,**C**,**E**); photos on right side show PAS-positive staining, magnification 200× (**B**,**D**,**F**). C—control rats (healthy rats), DM—diabetic rats, DM + PP—diabetic rats treated with Blue Congo extract for 14 days (at a dose of 165 mg/kg b.w./day).

We observed no signs of liver damage. In the DM and DM + PP group, there was an increased number of hepatocytes with two or more nuclei (Figure 8C,E). In both groups, an increased number of cells with PAS-positive reaction were noted. The content of PAS-positive cells in the DM + PP group was slightly lower than in the DM group (Figure 8D,F). However, in morphometrical analysis, these changes were irrelevant. No increased blood, leukocyte infiltration, or activation of fibroblasts was observed. In terms of toxicity, the liver is believed to be the main target organ which is used to metabolize foreign substances present in the intestines to other compounds whose hepatoxic effect is, in turn, impossible to predict [57].

## 3. Materials and Methods

### 3.1. Materials

Streptozotocin, 5,5′-dithiobis (2-nitrobenzoic acid), thiobarbituric acid, trichloroacetic acid, 2,4-dinitrophenylhydrazine, and chlorogenic acid were delivered from Sigma–Aldrich (St. Louis, MO, USA). Acetonitrile for liquid chromatography–mass spectrometry was purchased from POCh (Gliwice, Poland). Petunidin 3-*O*-glucoside chloride was purchased from Extrasynthese (Lyon Nord, France). All other reagents were of analytical grade.

### 3.2. Preparation of Extract from Blue Congo

The tubers of Blue Congo potatoes were obtained from the collection of the in vitro gene bank for potatoes in Bonin (Plant Breeding and Acclimatization Institute, National Research Institute, Bonin Research Center, Bonin, Poland).

Purple potatoes (100 g) were washed with running tap water, cut into pieces of approximately 2–3 cm, and ground down with a blender (First Austria, Vienna, Austria), forming pulp. Then, the potatoes mush was placed into a 500-mL flask containing 300 mL of aqueous ethanol (70%) and HCl (0.1%, *v/v*) and extracted for 30 min at 35 °C in a sonicator water bath (20 kHz, Sonic, Rho Italia). Next, the supernatant was filtered. Hydrochloric acid was added in order to prevent the degradation of the non-acylated compounds [58]. The extraction process by sonication was repeated three times in total. The obtained extract was spun for 15 min in a centrifuge (3500 rpm) at room temperature and then the ethanol was evaporated using a rotary evaporator (IKA RV 05B, Staufen, Germany) at a temperature not exceeding 40 °C. After evaporation of ethanol, the aqueous layer was loaded on an Amberlite^®^ resin (XAD-7) column (Sigma-Aldrich St. Louis, MO, USA), preconditioned with water. The extract rich in anthocyanins and phenolic acids was obtained after washing the column with 80% ethanol. The collected fraction was evaporated in a vacuum evaporator and dried using a heated air drier (at 35 °C) until dry mass. The obtained extract was stored at temperature of −30 °C until assayed. To produce 1 g of Blue Congo extract, 1 kg of raw potatoes was used (extraction efficiency = 0.1%).

### 3.3. Identification and Quantification of Compounds of Blue Congo Extract Using the UPLC–qTOF-MS/MS and HPLC–Photodiode Array (PDA) Methods

The method was previously described by Kucharska et al. [59]. The HPLC analysis was performed using a Dionex (Germering, Germany) system, equipped with the diode array detector model Ultimate 3000, quaternary pump LPG-3400A, autosampler EWPS-3000SI, and thermostated column compartment TCC-3000SD, and controlled by Chromeleon v.6.8 software (Thermo Scientific Dionex, Sunnyvale, CA, USA). The Cadenza Imtakt column C5-C18 (75 × 4.6 mm, 5 µm) was used. The mobile phase was composed of solvent A (4.5% aqueous formic acid, *v/v*) and solvent B (100% acetonitrile). The elution system was as follows: 0–1 min 5% B in A, 20 min 25% B in A, 21 min 100% B, 26 min 100% B, and 27 min 5% B in A. The flow rate of the mobile phase was 1.0 mL/min, and the injection volume was 20 µL. Anthocyanins were detected at 520 nm, while phenolic acids and their derivatives were detected at 320 nm. Anthocyanins were expressed as petunidin-3-*O*-glucoside equivalent, while phenolic acids and derivatives were expressed as chlorogenic acid equivalent. The results were expressed as mg/g d.m.

The UPLC–qTOF-MS/MS method was previously described by Mizgier et al. [60]. Compound identification was performed on an Acquity ultraperformance liquid chromatography (UPLC) system coupled with a quadrupole time-of-flight (Q-TOF) MS instrument (UPLC/Synapt Q-TOF MS, Waters Corp., Milford, MA, USA) with an electrospray ionization source (ESI). Separation was achieved on an Acquity TM BEH C18 column (100 mm × 2.1 mm inner diameter (i.d.), 1.7 µm; Waters Corporation, Milford, MA, USA). The detection wavelength was set at 520 nm. The mobile phase was a mixture of 42% formic acid (A) and 100% acetonitrile (B). The gradient program was as follows: initial conditions—99% (A), 12 min—75% (A), 12.5 min—100% (B), 13.5 min—99% (A). The flow rate was 0.45 mL/min, and the injection volume was 5 µL. The column was operated at 30 °C. Major operating parameters for the Q-TOF MS were set as follows: capillary voltage, 2.0 kV; cone voltage, 45 V; cone gas flow, 11 L/h; collision energy, 30 eV; source temperature, 100 °C; desolvation temperature, 250 °C; collision gas, argon; desolvation gas, nitrogen; flow rate, 600 L/h; data acquisition range, *m/z* 100–1000 Da; ionization mode, positive (anthocyanins) and negative (phenolic acids). The data were collected using Mass-Lynx TM V 4.1. software (Waters Corporation, Milford, MA, USA).

### 3.4. Experimental Animals

Wistar rats (male, *n* = 32, eight weeks old, 135 ± 8 g) were used in the study. Animals were handled in accordance with the Ethics Committee of the Department of the Faculty of Biology (Protocol N 2, 24 October 2018), Ivan Franko National University of Lviv, Ukraine, and complied with the Directive 2010/63/EU of the European Parliament and the Council of 22 October 2010 on the protection of animals used for scientific purposes and the National Institutes of Health guide for the care and use of laboratory animals (NIH Publications No. 8023, revised 1978). Animals had free access to a standard chow and water and were kept under a 12 h light/dark cycle. The room temperature and humidity were maintained automatically at about 21 ± 2 °C and 55 ± 5%, respectively.

### 3.5. Induction of Diabetes Mellitus in Rats

Diabetes mellitus was induced by intraperitoneally injecting the rats with STZ (Sigma-Aldrich, St. Louis, MO, USA) at a dose of 55 mg/kg body weight (b.w.) after two weeks of adaptation. STZ was freshly prepared as solution in 10 mM sodium citrate buffer (pH 4.5) and injected to fasted overnight animals. On the third day after the STZ injection, the blood was sampled from the tail vein of STZ-injected animals, and glucose levels were measured using the glucose oxidase method (Filisit diagnostics kit, Dnipro, Ukraine) and glucometer ABRA (Diagnosis S.A., Białystok, Poland). Rats having a blood glucose concentration over 12 mmol/L were considered to have type 1 diabetes and used for further experiments. The control group was selected among intact animals which had glucose concentrations in the range 5.0–5.8 mmol/L. The rats were divided into four experimental groups containing eight rats each: group 1 (C)—control rats (healthy animals), group 2 (C + PP)—control rats receiving extract from Blue Congo, group 3 (DM)—rats with STZ-induced DM, group 4 (DM + PP)—diabetic rats treated with extract from Blue Congo. The treatments were given by oral administration to rats (1 mL/rat) using rat gavage for a period of 14 days. Rats from the second and fourth experimental groups were orally dosed with the Blue Congo extract in amounts of 165 mg/kg b.w./day. The administration dose was selected on the basis of previous studies with red cabbage extract rich in anthocyanins [61] and the content of total polyphenols and anthocyanins of Blue Congo extract.

### 3.6. Oral Glucose Tolerance Test 

The oral glucose tolerance test (OGTT) was performed in rats after 14 days of treatment with the Blue Congo extract. Before the OGTT, rats were fasted for 17 h. Blood samples were collected from the tail vein at 0 min and at 15, 30, 60, 90, and 120 min after the administration of glucose solution (1 g/kg b.w.). The index of the area under the glycemic curve (AUC_glc_) was calculated in order to show the general increase in glucose concentrations after glucose consumption.

### 3.7. Collection of Blood and Preparation of Samples

After the last treatment, rats were fasted overnight and were anesthetized using diethyl ether and euthanized by decapitation. Blood was collected into vials with heparin. Then, 2 mL of blood was centrifuged at 3000 rpm to obtain plasma. Plasma was frozen and stored at −20 °C for further measurements. Erythrocytes were washed three times with cold (4 °C) phosphate-buffered saline (PBS, pH 7.4). Leukocytes were isolated from blood by centrifugation in ficoll-triombrast density (*r* = 1.076–1.078). Afterward, the cells were washed three times with cold (4 °C) PBS.

### 3.8. Erythrocyte- and Leukocyte-Related Parameters

The content of glycated hemoglobin (HbA1c) in blood was measured following the method described by Vitak et al. [62], based on the generation of 5-hydroxymethylfurfural from ketoamine after treatment with oxalic acid and its subsequent reaction with thiobarbituric acid to form a colored adduct. Hemoglobin concentrations in the whole blood were determined using the cyanmethemoglobin method, whereas mean cell hemoglobin (MCH) was determined according to Souza et al. [63]. The total number of erythrocytes and leukocytes in blood was calculated manually using a counting chamber.

### 3.9. Assessment of Antioxidant Activities

#### 3.9.1. Superoxide Dismutase 

The activity of leukocyte superoxide dismutase (SOD, EC 1.15.1.1) was determined by the method based on the restoration of nitrotetrazolium using a superoxide radical. SOD activity was analyzed following the method described by Kakkar et al. [64] with modification. One unit of enzymatic activity was defined as the amount of enzyme causing 50% inhibition of the reduction to formazan observed in the blank. The absorbance of the reaction mixture was read at λ = 540 nm against the blank. The results were expressed as U/mg of protein.

#### 3.9.2. Catalase

Catalase (CAT, EC 1.11.1.6) activity was measured by decreasing the color intensity of the complex forming H_2_O_2_ with molybdenum salts [65]. The reaction mixture consisted of 0.05 M Tris-HCl buffer (pH 7.8), a 0.03% solution of H_2_O_2_, and the test sample. The reaction was stopped by adding a 4% solution of ammonium molybdate after 10 min of incubation. Measurements were carried out by spectrophotometry at a wavelength of λ = 410 nm. One unit of CAT activity was calculated as one nmol of H_2_O_2_/min × mg of protein.

#### 3.9.3. Glutathione Peroxidase

Glutathione peroxidase (GPx, EC 1.11.1.9) activity was assessed by the rate of oxidation of reduced glutathione before and after incubation with tertiary butyric hydroperoxide by color reaction with 5,5′-dithiobis (2-nitrobenzoic acid) (DTNB, Ellman’s reagent, Acros Organics, Geel, Belgium) [66]. The reaction mixture contained Tris-HCl buffer 0.1 M (pH 8.5), a 4.8 mM solution of reduced glutathione (GSH), 20 mM tert-butyl hydroperoxide, trichloroacetic acid (20%), 0.01 M Elman reagent, and the tested sample. The intensity of the color of control and study samples was determined spectrophotometrically at a wavelength of λ = 410 nm against the blank. GPx activity was expressed in μmol/min × mg of protein.

#### 3.9.4. Reduced Glutathione 

Reduced glutathione (GSH) was measured following the method described by Rizvi et al. [67], based on the property of GSH to reduce DTNB, forming a yellow-colored anionic product whose optical density was measured at λ = 412 nm by spectrophotometry. A molar extinction coefficient of 13,600 was used for the nitrobenzoate ion. Concentration of GSH was expressed in nmol/mg of protein. 

### 3.10. Lipid Peroxidation

Malondialdehyde (MDA) in blood plasma was assayed by using a thiobarbituric acid (TBA) reactive substance at high temperature, forming a colored complex, expressed as nanomoles per milliliter plasma with a molar absorption coefficient of 156,000 M^−1^·cm^−1^ at λ = 532 nm [66]. The reaction mixture consisted of 10 mM phosphate buffer (pH 7.4), 1 mM potassium permanganate, and the tested sample. Firstly, 10 mM ferrous sulfate was added twice to start the reaction. Then, 20% trichloroacetic acid was added to stop the reaction. Finally, malondialdehyde (MDA) reacted with thiobarbituric acid, forming a colored product.

### 3.11. Assay of Advanced Oxidation Protein Products

Determination of AOPPs was based on spectrophotometric detection, as described by Kalousova et al. [49]. Firstly, 200 μL of blood plasma diluted 1:5 with PBS, 200 μL of chloramine-T standard solution (0 to 100 µmol/L) for calibration, and 200 μL of PBS as blank were placed in a microtiter plate, followed by the addition of 10 μL of 1.16 M potassium iodide (KI) and 20 μL of acetic acid. The absorbance of the reaction mixture was read at λ = 340 nm against the blank. Concentration of AOPPs was expressed in chloramine units (µmol/g of protein).

### 3.12. Assay of Advanced Glycation End Products

The amount of blood plasma AGEs was determined following the method described by Putta and Kilari [68]. Plasma was diluted in a 1:50 ratio with PBS (pH 7.4). Bovine serum albumin preparation (1 mg/mL of 0.1 N NaOH) was used as a standard, and its fluorescence intensity was defined as one unit of fluorescence (AU). The level of AGEs in plasma was determined by measuring the fluorescence at an excitation wavelength of λ = 370 nm and emission wavelength of λ = 440 nm. The fluorescence intensity of the samples was expressed as AU per mg of protein.

### 3.13. Assay of Oxidative Modified Proteins

The method for determining the level of OMPs was based on spectrophotometric detection of the reaction of aldehyde and ketone groups of aliphatic amino acid residues with the 2,4-dinitrophenylhydrazine reagent to form protein-conjugated 2,4-dinitrophenylhydrazones with a characteristic absorption spectrum at λ = 370 nm (OMP_370_—OMPs of neutral characteristics) and at λ = 430 nm (OMP_430_—OMPs of basic characteristics). Determination of the level of OMPs was measured following the method described by Demkovych et al. [69] with a slight modification. The reaction mixture consisted of 0.85% sodium chloride, 0.1 M 2,4-dinitrophenylhydrazine, 2 M hydrochloric acid, 10% trichloroacetic acid, 8 M urea solution, and the tested sample. The results of OMP_370_ were expressed using a molar absorption coefficient of 22,000 M^−1^·cm^−1^, and the results of OMP_430_ were expressed as conditional units (c.u.) of protein carbonyl groups per gram of protein without a molar absorption coefficient.

### 3.14. Histopathology Study

Tissue samples obtained from liver, kidney, and muscles were fixed in 4% buffered formalin of pH 7.2 for three days, washed in tap water, dehydrated in a series of alcohols, and embedded in paraffin. Paraffin sections (4 µm thick) were cut and stained with hematoxylin–eosin (H&E, Sigma–Aldrich, St. Louis, MO, USA) and stained with periodic acid–Schiff (PAS reaction kit, Sigma–Aldrich, St. Louis, MO, USA). Histopathological changes in the liver, kidney, and muscles were visualized using a Nikon Eclipse 80i microscope (NIKON, Amsterdam, Netherlands) supported by NIS-Elements Ar software for morphometrical analysis. Morphometrical analysis of the muscle thickness, thickness of the basal membrane of the proximal tubule, and the number of PAS-positive hepatocytes was carried out using Statistica 12.0 software (StatSoft, Kraków, Poland).

### 3.15. Statistical Analysis

Data are shown as mean values ± standard deviation (SD). The results were analyzed by one-way ANOVA followed by Duncan′s test. In this study, *p*-values of 0.05, 0.01, and 0.001 were considered statistically significant. The program Statistica 12.0 was used for all statistical calculations. Designations were as follows: * *p* < 0.05, ** *p* < 0.01, *** *p* < 0.001 compared to the control group; # *p* < 0.05, ## *p* < 0.01, ### *p* < 0.001 compared to the DM group.

## 4. Conclusions

To the best of our knowledge, this is the first report related to the antidiabetic and antioxidant properties of purple potato extract from the Blue Congo variety in STZ-induced diabetic rats. The study showed that the predominant compound identified in the extract from Blue Congo is the acylated anthocyanin petunidin-3-*O*-*p*-coumaryl-rutinoside-5-*O*-glucoside, which accounted for about 74% of the total anthocyanin content. Administration of Blue Congo extract for 14 days to STZ-induced diabetic rats improved the activities of antioxidant enzymes, and effectively attenuated the formation of oxidative stress markers that reflected the antioxidant potency of the extract. Oxidative stress inhibition by Blue Congo was expressed through increased levels of GSH and activity of antioxidant enzymes (SOD, CAT, GPx) in leukocytes. In addition, we noticed a decrease in the level of MDA, AOPPs, and AGEs, as well as that of oxidative modified proteins (with neutral and basic characteristics) in rats′ blood plasma after administration of the Blue Congo extract. These antioxidant activities were coupled with anti-diabetic effects, as assessed by decreased fasting blood glucose and glycated hemoglobin levels in diabetic rats after administration of the extract. Overall, the study demonstrated the potential application of Blue Congo extract to reinstate diabetes-induced oxidant–antioxidant imbalance of peripheral blood. Our preliminary research can be helpful for developing and testing nutraceuticals for people suffering from diabetes and its complications.

## Figures and Tables

**Table 1:** Characterization and content (mg/g dry matter (d.m.)) of main compounds of Blue Congo extract. Rt—retention time.

Peak No.	Compound	Rt (min)	[M − H]^+^/[M + H]^−^ (*m/z*)	MS/MS Fragments (*m/z*)	Content (mg/g d.m.)
**Anthocyanins**					
1	Petunidin-3-*O*-rutinoside-5-*O*-glucoside	3.52	787.2288^+^	479.1120/317.0676	4.42 ± 0.23
2	Petunidin-3-*O*-*p*-caffeyl-rutinoside-5-*O*-glucoside	6.50	949.2610^+^	787.2059/479.1165/317.0676	7.41 ± 0.33
3	Delphinidin-3-*O*-*p*-coumaroyl-rutinoside-5-*O*-glucoside	6.68	919.2560^+^	757.2056/465.1026/303.0494	2.79 ± 0.24
4	Petunidin-3-*O*-*p*-coumaryl-rutinoside-5-*O*-glucoside	7.19	933.2700^+^	771.2118/479.1210/317.0676	53.95 ± 2.55
5	Malwidin-3-*O*-*p*-coumaryl-rutinoside-5-*O*-glucoside	7.69	947.2831^+^	785.2213/493.1294/131.0825	4.10 ± 0.19
**Phenolic Acid**					
1	3-*O*-Caffeoylquinic acid (neochlorogenic acid)	3.38	353.0338^−^	191.0562/179.0342	24.22 ± 1.03
2	4-*O*-Caffeoylquinic acid (cryptochlorogenic acid)	4.59	353.0915^−^	173.0458/179.0342/191.0534	29.69 ± 1.33
3	Methyl-3-caffeoylquinate	4.70	367.1057^−^	161.0237	1.81 ± 0.11
4	5-*O*-Caffeoylquinic acid (chlorogenic acid)	4.85	353.0838^−^	707.1813/191.0563	98.65 ± 4.74
5	Methyl-4-caffeoylquinate	5.63	367.1051^−^	161.0237	6.64 ± 0.26
6	Methyl-5-caffeoylquinate	6.41	367.1057^−^	179.0342/161.0237/135.0450	6.39 ± 0.31
**Total**	**Anthocyanins and phenolic acid**				**237.07 ± 11.32**

Data are expressed as means ± SD, *n* = 3. Anthocyanins are expressed as petunidin-3-*O*-glucoside equivalents; phenolic acids are expressed as chlorogenic acid equivalents.

**Table 2:** The effect of Blue Congo extract on the number of leukocytes and red blood cells, and hemoglobin concentration in rats′ blood. Designations: ** *p* < 0.01 compared to the control group; ^#^ *p* < 0.05 compared to the DM group; C—control; PP—treated with purple potato extract; DM—diabetes mellitus.

Parameters	Groups			
	C	C + PP	DM	DM + PP
Number of leukocytes, 10^3^ µL^−1^	10.14 ± 0.84	14.00 ± 0.63**	11.17 ± 0.70	12.15 ± 1.11
Number of red blood cell, 10^6^ μL^−1^	5.48 ± 0.13	5.08 ± 0.23	6.06 ± 0.32	6.24 ± 0.41
Hemoglobin content, g%	15.00 ± 0.84	12.86 ± 0.56	15.75 ± 0.79	16.00 ± 0.44
Mean cell hemoglobin, pg	25.53 ± 0.77	25.50 ± 1.00	20.40 ± 1.67	25.58 ± 1.66^#^
Color index, c.u.	0.77 ± 0.02	0.77 ± 0.03	0.62 ± 0.05	0.79 ± 0.05^#^
